# Supplementary material for: Unpacking the asymmetric impact of exchange rate volatility on trade flows: A study of selected developed and developing Asian economies
Source: PLoS One. 2023 Oct 11;18(10):e0291261. doi: 10.1371/journal.pone.0291261 (PMC10566717; doi:10.1371/journal.pone.0291261)
Supplement: S1 Appendix — (DOCX) [file pone.0291261.s001.docx]

**APPENDIX A.**

**Data Definition and Sources**

All the secondary data has been taken from the following sources for a period 1980I to 202IV.

WITS (World Integrated Trade Solution)

IFS (International Financial Statistics)

Variables

X- Total export of each country to the world. The unit value of exports deflates from nominal exports. The data is from source b.

M- Total imports of each country from the world. The unit value of imports deflates nominal imports. The information is from source b.

Y- Measure the domestic income in every country. We followed Bergstrom [46] and produced quarterly data in the absence of any country's quarterly statistics. We utilized CPI in place of a GDP deflator because none of the countries had one. All information is from source b.

YW- The total of the real GDP of the USA and the OECD nations serves as a proxy for global real income. A and B are the sources of the data.

REX- Real exchange rate. Data from source b is used. Interpolation is used to fill in missing periods.

V- volatility measure of exchange rate. We construct this measure using a Generalized Autoregressive Conditional Heteroskedasticity (GARCH 1, 1) method, as employed by Bahmani-Oskooee and Aftab [47].
