# Supplementary material for: Unpacking the asymmetric impact of exchange rate volatility on trade flows: A study of selected developed and developing Asian economies
Source: PLoS One. 2023 Oct 11;18(10):e0291261. doi: 10.1371/journal.pone.0291261 (PMC10566717; doi:10.1371/journal.pone.0291261)
Supplement: Attachment — Submitted filename: Response to reviewers comments 26-06-2023.docx [file pone.0291261.s002.docx]

**Unpacking the Asymmetric Impact of Exchange Rate Volatility on Trade Flows: A Study of Selected Developed and Developing Asian Economies**

**PONE-D-23-08701**

**Response to the Editor / Referees**

Dear Editor/Referee,

Thank you very much for reviewing our manuscript, encouraging comments, constructive feedback, and valuable suggestions. We have worked diligently to address each of your remarks and suggestion and are resubmitting a substantially improved version of the paper. We include below your comments in italics, and our responses follow. Please also see the changes in yellow in the revised version of the paper.

**Referee 1**

1. *The abstract should be rewritten by adding methodology and policy implications emerging from this study.*

Response: Thank you for your valuable comments. We have rewritten the abstract. In the abstract, we have incorporated the objective of the study, the methodology applied, major findings, and implications of our study.

2. *The introduction section should be narrating the significance of the topic and rationale of the study*

Response: Thank you very much for identifying one important weakness of our initial submission. Now, we have incorporated the significance of the study and added new citations from the PLOS ONE journals.

3. *The literature review section is weak and needs to be added to relevant and recent literature.*

Response: Thank you for your valuable comments. We have revised and added some new citations in the literature section.

4. *The methodological section is good written, however, theoretical framework and choice of the variables in the model are missing. For this purpose, following article will serve the purpose to some extent:*

Response: Thank you very much for this comment which helps us to revisit our initial understanding. In our revised submission, we have included a revised methodology and explained the Model in detail.

5. *The study uses GARCH model for volatility measurement. There are many other methods why only the GARCH is preferred on others, Justify.*

Response: Thank you very much for this comment which helps us to revise our initial understanding. In our revised submission, we have included the logic of why we used GARCH model and why we are not using other methods in this study.

6. *The choice of the sample countries should be based on some logic. It should be justified.*

Response: Thank you very much. Now, we have incorporated justifications behind the selection of the Asian developed and developing countries (page 7).

7. *Mostly, the results of the coefficients estimate for different countries are different. The differences should be explained logically.*

Response: Thank you very much. We have revised the equations that have wrong coefficient signs We also revised the interpretations of the results and add why we used asymmetry.

**Referee 2**

1. *The manuscript lacks coherence, there is large amount of irrelevant literature in introduction section that is not in line with objectives.*

Response: Thank you very much for encouraging comments regarding our work. We are happy and delighted to receive such appreciation from learned reviewer. We have revised our objective of the study, and revised introduction part and also add some new citations which is coherent with our objectives.

2. *The equations in methodology section are not clear because there are no proper subscripts.*

Response: Thank you very much for identifying one of the major weaknesses of our initial submission. We have deployed our best effort to improve the methodology section. Now we have changed the equations and corrected the values of the coefficients.

3. *Authors have not provided any justification for using the selected developing and developed countries.*

Response: Thank you very much for this comment which helps us to revisit our initial understanding. In our revised submission, we have included the justification for why we have selected Asian developing and developed nations in this study (page 7).

4. *The findings and conclusions are not in line with the objectives given in the introduction section.*

Response: Thank you for this helpful comment. We have revised our study objective and rewrite the conclusion of the study. And on the bases of our findings, we also add some policy recommendations.

In conclusion, we are thankful to you for your constructive feedback on our submission, which has improved the paper significantly. We have tried our best to address the comments in our revised version and believe that the paper has been changed substantially. We will be waiting for your valuable comments on our revised submission, please.
